# Strigolactones Control Root System Architecture and Tip Anatomy in *Solanum lycopersicum* L. Plants under P Starvation

**DOI:** 10.3390/plants9050612

**Published:** 2020-05-11

**Authors:** Veronica Santoro, Michela Schiavon, Francesco Gresta, Andrea Ertani, Francesca Cardinale, Craig J. Sturrock, Luisella Celi, Andrea Schubert

**Affiliations:** 1 Dipartimento di Scienze Agrarie, Forestali e Alimentari (DISAFA), Largo Paolo Braccini 2 (già Via Leonardo da Vinci, 44), 10095 Grugliasco (Torino), Italy; veronica.santoro@unito.it (V.S.); francesco.gresta@unito.it (F.G.); andrea.ertani@unito.it (A.E.); francesca.cardinale@unito.it (F.C.); luisella.celi@unito.it (L.C.); andrea.schubert@unito.it (A.S.); 2 Dipartimento di Agronomia, Animali, Alimenti, Risorse naturali e Ambiente (DAFNAE), Viale dell’Università 16, 35020 Legnaro (Padova), Italy; 3 School of Biosciences, University of Nottingham, Loughborough LE12 5RD, UK; Craig.Sturrock@nottingham.ac.uk

**Keywords:** phosphorus, strigolactones, root architecture, root anatomy, tomato

## Abstract

The hormones strigolactones accumulate in plant roots under phosphorus (P) shortage, inducing variations in plant phenotype. In this study, we aimed at understanding whether strigolactones control morphological and anatomical changes in tomato (*Solanum lycopersicum* L.) roots under varying P supply. Root traits were evaluated in wild-type seedlings grown in high vs. low P, with or without exogenous strigolactones, and in wild-type and strigolactone-depleted plants grown first under high vs. no P, and then under high vs. no P after acclimation on low P. Exogenous strigolactones stimulated primary root and lateral root number under low P. Root growth was reduced in strigolactone-depleted plants maintained under continuous P deprivation. Total root and root hair length, lateral root number and root tip anatomy were impaired by low strigolactone biosynthesis in plants grown under low P or transferred from low to no P. Under adequate P conditions, root traits of strigolactone-depleted and wild-type plants were similar. Concluding, our results indicate that strigolactones (i) control macro- and microscopic changes of root in tomato depending on P supply; and (ii) do not affect root traits significantly when plants are supplemented with adequate P, but are needed for acclimation to no P and typical responses to low P.

## 1. Introduction

Strigolactones are a group of carotenoid-derived compounds [1,2] whose presence has been confirmed in a broad variety of plant species, including monocots, dicots, and ancestral plants [3,4]. Strigolactone biosynthesis takes place mainly in the roots, where they are produced at extremely low concentrations, i.e., in the pico- and nanomolar range, usually as a blend of molecules that is typical of the species. The core biosynthetic module comprises an isomerase (D27) and two carotenoid cleavage dioxygenases (CCD7 and CCD8) that sequentially act to convert β-carotene to carlactone. Downstream of carlactone, the pathway diversifies in different species, but one or more cytochrome P450s along with oxidase(s) are thought to further convert carlactone to different strigolactones [5].

Originally, strigolactones were discovered as stimulatory compounds for seed germination of root parasitic plants, such as *Striga*, after which they are named [6]. Later, they were recognized as pivotal host detection signals for symbiotic arbuscular mycorrhizal (AM) fungi [7]. Only more recently, strigolactones have been classified as a new class of plant hormones with key regulatory roles in development [8]. Specifically, strigolactones can inhibit shoot branching and reproductive maturity, promote leaf senescence and affect seed germination [9,10]. They were also suggested to promote primary root growth, lateral and adventitious root formation and root hair development, but with differences among plant species. Notably, in the model plant *Arabidopsis thaliana*, their role has been recently disentangled from that played by the sibling pathway dependent on KARRIKIN INSENSITIVE2 (KAI2) action [11]. The KAI2 receptor and its unidentified strigolactone-like ligand seem to be ultimately responsible for the regulation of root hair length and density under normal growth conditions, while strigolactones regulate lateral root density [11]. Whether this is true also in other species is not known at present. Because of their inducibility by low phosphorus (P) and, with exceptions, by low nitrogen, strigolactones have been long thought to be involved in the responses to nutrient deprivation, among other abiotic stresses [1,10,12,13,14]. Phosphorus deficiency can also stimulate strigolactone exudation from the roots; this, in turn, is suggested to promote the establishment of AM symbioses and thus, indirectly, relieve P stress [13].

Phosphorus is one of the essential macronutrients required by plants for their growth and development [15], being a structural component of key biomolecules [16,17] and taking part in primary cellular metabolic processes [15,18,19]. Therefore, whole-plant growth is substantially reduced or inhibited by P limitation [2]. It is noteworthy that P deficiency represents one of the major restraints in agricultural production, as P in soil is one of the most immobile, inaccessible, and unavailable among all nutrient elements [17,20]. Phosphorus is absorbed and assimilated by plants as phosphate (Pi), which occurs at fairly low concentrations in the soil solution, typically in the 1–10 µM range [16,21,22,23]. This is because Pi tends to establish strong interactions with soil colloids (mainly Fe and Al oxides), on which it can be almost irreversibly adsorbed or occluded [24]. The concentrations of available P in soil can thus range from null to values that remain anyway well below the critical level needed for optimal plant growth, which corresponds to tens of µM for the most demanding species [23]. Different studies have underlined how slight variations of P concentration during plant growth can bring about evident differences in the overall plant development [25]. Dramatic effects on root architecture are reported, for instance, when P available for plant uptake is lower than 50 µM [26]. These effects are apparently dependent on the intensity of P deficiency [27,28].

Previous studies have indicated a major role for strigolactones as signaling molecules able to trigger morphological, physiological and biochemical responses associated with plant acclimation to P deficiency conditions [9,14,29]. In P-starved *A. thaliana* plants especially, strigolactones have been proposed to suppress bud outgrowth and shoot branching to reduce internal P utilization, while initial reports have suggested that strigolactones promote lateral root development and root hair formation to increase the root surface area in contact with soil, while inhibiting the primary root growth [9,30]. In light of recent reassessment of the strigolactone role in shaping root morphology under non-stressful conditions, as mentioned above [11], these findings would require further validation both in *A. thaliana* and in other plants for which the appropriate genetic tools are available.

Tomato (*Solanum lycopersicum* L.), beyond being a valuable crop worldwide, has become an important model species for research on strigolactones. The blend of the major strigolactones produced by this species has been determined [31,32] and the prompt increase of biosynthesis in roots in response to P starvation is confirmed [31,33]. Furthermore, the shoot phenotype of strigolactone-depleted tomato plants is consistent with the conserved role of strigolactones in development [34,35,36]. However, whether strigolactones affect morphological adjustments to P deprivation in tomato has not yet been thoroughly investigated.

Based on the previous considerations, we hypothesized that the effect of P stress on root architecture and morphology may be mediated by strigolactones in tomato. Therefore, the current study aims at understanding the impact of strigolactones on macro- and microscopic root features, as a response to P starvation in this plant. We first investigated the effect of exogenous strigolactone on wild-type plants under moderate P deprivation, and further contrasted a wild-type with a strigolactone-depleted transgenic line, assessing primary and lateral root growth under conditions of continuous P deprivation — either complete or moderate. We then assessed the effects of progressively decreasing P supply, to better mimic P availability to plants in field conditions. Finally, we studied in detail the morphology and root tip anatomy of these genotypes under progressive P deficit. Based on our results, we conclude that strigolactones may mediate macroscopic root architecture changes induced by P deprivation, enhancing the traits that generally account for better soil exploration, such as root number, length and volume. Additionally, strigolactones seem responsible for changes in root anatomy, which relate to adjustments in root development under P shortage.

## 2. Results

### 2.1. Exogenous Strigolactones Increase Primary Root Length and Lateral Root Number under Continuously Low P Availability

**Figure 1:**
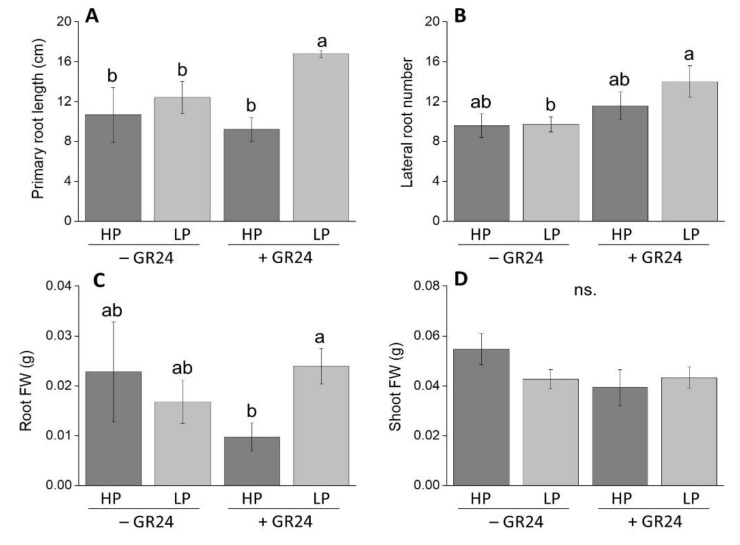
Main biometrics of two-week-old wild-type (WT) seedlings grown either in a standard MS (high P, HP) or in Pi-deprived conditions (low P, LP), with (+ GR24) or without (− GR24) 5 µM *rac*-GR24: (**A**) primary root length; (**B**) number of lateral roots; (**C**) root fresh biomass; (**D**) shoot fresh biomass. Each column represents the average of three (**C**,**D**) to six seedlings (**A**,**B**) with standard error. Statistical significance of differences between means is indicated by different letters above bars (*p* ≤ 0.05).

As a first step to assess whether strigolactones affect root morphology depending on the P status, we scored root biometrics on M82 seedlings grown in vitro in the presence or absence of 5 µM exogenous strigolactone (in the form of the synthetic analogue *rac*-GR24), under high and low P conditions. No significant differences in primary root length were observed following treatment with *rac*-GR24 in high P seedlings; conversely, *rac*-GR24 treatment significantly increased primary root length of low-P seedlings (Figure 1A). The lateral root number also increased in *rac*-GR24-treated seedlings only at low P (Figure 1B). Accordingly, root fresh weight increased following strigolactone treatment at low P compared to high P, *rac*-GR24-treated seedlings, suggesting that the effects of *rac*-GR24 treatment on root diameter depend on the P status (Figure 1C). No appreciable changes in shoot biomass were observed under any condition (Figure 1D).

### 2.2. Strigolactone Depletion Alters Primary and Lateral Root Growth under Continously High or No P Conditions

**Figure 2:**
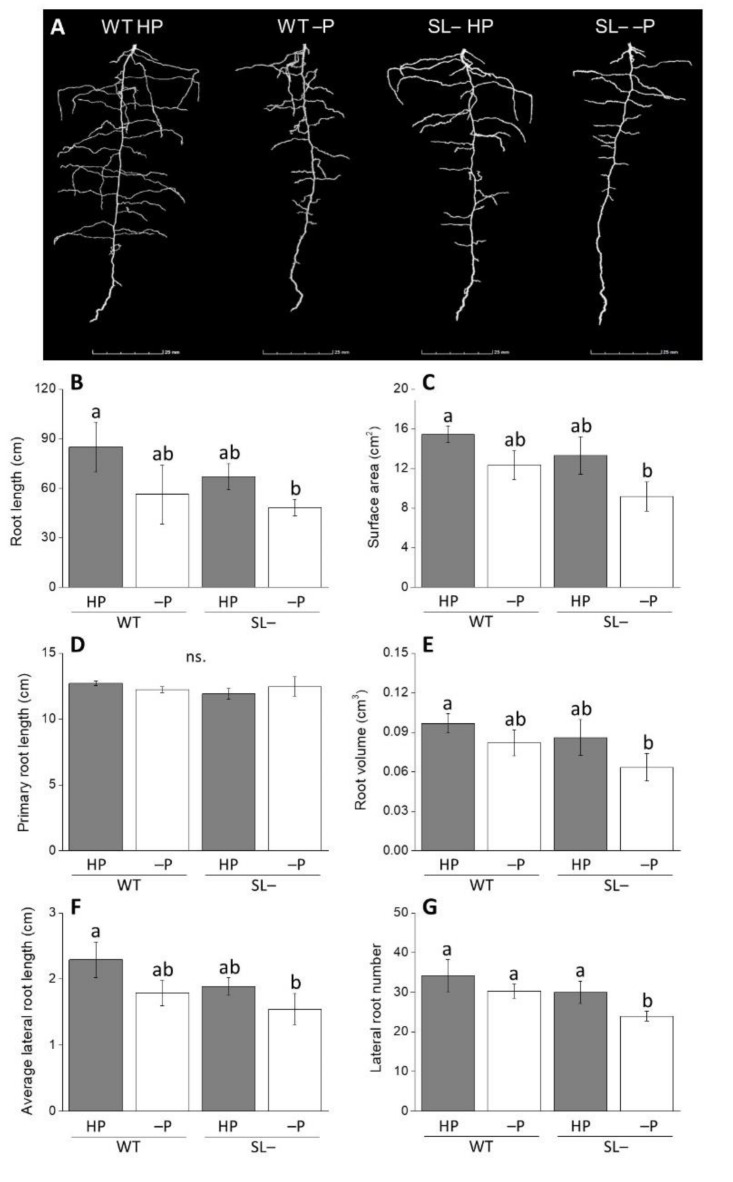
(**A**) Image of root architecture displayed by X-ray CT; (**B**–**G**) Root growth-associated parameters of wild-type (WT) and strigolactone-depleted plants (SL–) grown for 10 days inside columns filled with quartz sand and daily watered with modified Hoagland solution containing either high P (HP) or no P (–P). Data represent the means of measurements on 3 independent replicates per genotype and condition (± SE) obtained via X-ray CT. Different letters above bars indicate significant differences between treatments (*p* ≤ 0.05).

In order to confirm data obtained with exogenous strigolactones, we contrasted the response of a wild-type genotype (M82) with a strigolactone-depleted transgenic line grown under conditions of zero P (no P addition) and high P (Figure 2A) using X-ray computed tomography (CT). Differences in root traits were evident between wild-type and strigolactone-depleted plants, both under zero P and high P conditions (Figure 2B–G). Total root length (Figure 2B) strongly correlated with the average lateral root length (r^2^ = 0.995). Average lateral root length, root surface area, volume and lateral root number (Figure 2B,C,E–G) followed a similar trend, whereby impairment of strigolactone synthesis weakly decreased these parameters under both P regimes. On the contrary, in this experimental set up, primary root length was not affected by impairment of strigolactone biosynthesis under either P supply status (Figure 2D).

### 2.3. Strigolactone Depletion Affects Root System Architecture of Plants Grown under Increasingly Severe P Starvation

Constant P provision or sudden P unavailability may not be common conditions for plants in field environments, as P availability in soils can be progressively reduced due to plant uptake or fixation processes, eventually leading to P deficiency. Thus we set up an experiment where wild-type and strigolactone-depleted plants were first grown in vitro for 10 days under low or high P supply; then high P plants were transplanted to an agar medium with the same P concentration, while low-P plants were transplanted onto agar medium containing no P.

**Figure 3:**
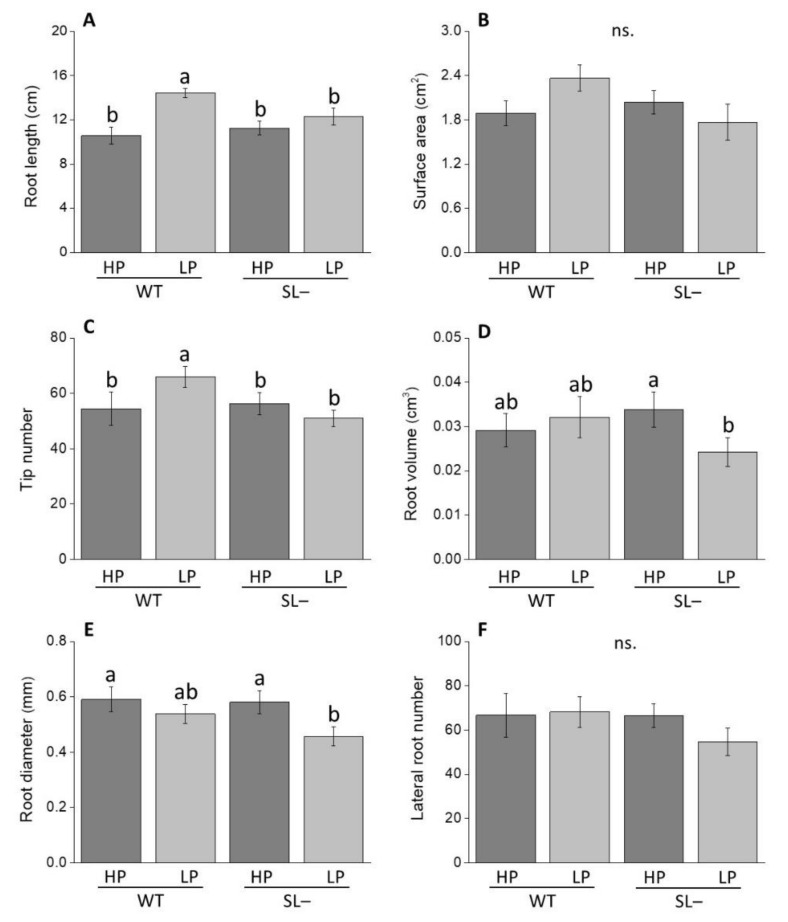
Root growth-associated parameters of wild-type (WT) and strigolactone-depleted plants (SL–) grown for 10 days in MS medium with either high P (HP) or low P (LP). (**A**) Root length; (**B**) surface area; (**C**) tip number; (**D**) root volume; (**E**) root diameter; (**F**) lateral root number. Data represent the means of 14 measurements per treatment (± SE) obtained via WinRHIZO. Different letters above bars indicate significant differences between treatments (*p* ≤ 0.05).

After 10 days of growth in low P, wild-type plants displayed higher values of total root length compared to strigolactone-depleted plants (Figure 3A). No differences in root length were observed between wild-type and strigolactone-depleted plants under high P supply. This trend was similar as for root surface area (although not significantly in this case, Figure 3B) and root tip number (Figure 3C). Root volume (Figure 3D) and diameter only significantly decreased in strigolactone-depleted plants under low P conditions with respect to high P (Figure 3E). No significant variation in lateral root number was revealed between plants under any conditions (Figure 3F).

**Figure 4:**
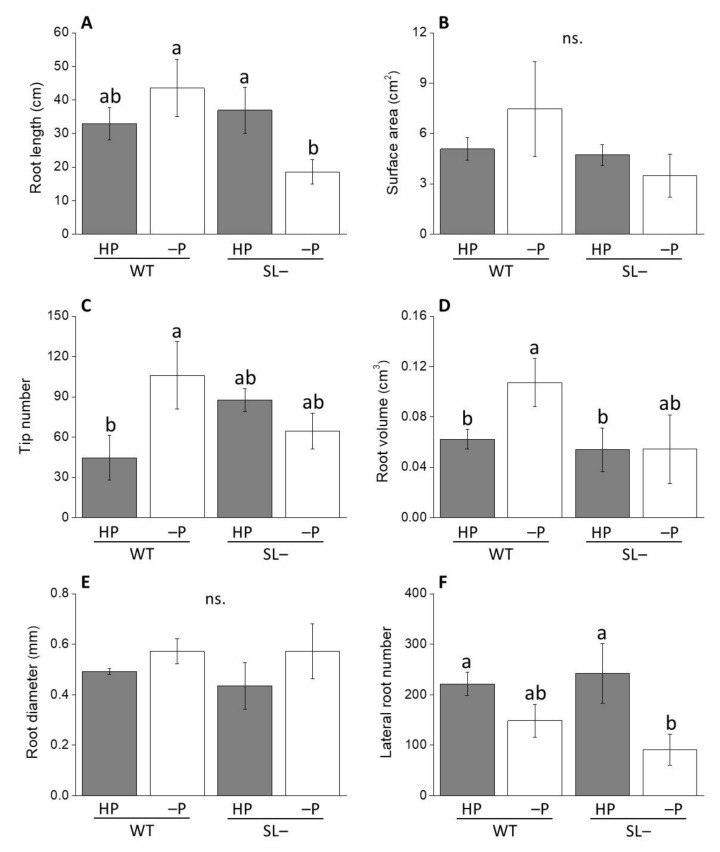
Root growth-associated parameters of wild-type (WT) and strigolactone-depleted plants (SL–) grown for 10 days in MS medium with either high P or low P and further transferred for one week to MS medium containing either high P (HP) or no P (–P), respectively. (**A**) Root length; (**B**) surface area; (**C**) tip number; (**D**) root volume; (**E**) root diameter; (**F**) lateral root number. Data represent the means of 3 measurements per treatment (± SE) obtained via WinRHIZO. Different letters above bars indicate significant differences between treatments (*p* ≤ 0.05).

When wild-type and strigolactone-depleted plants were transferred to new agar plates to either maintain high P or further decrease low P to no P status (hereby called zero P), differences in root traits became more prominent (Figure 4). Specifically, total root length of plants grown at zero P was substantially lower in strigolactone-depleted than in wild-type while still no differences were recorded between genotypes under high P (Figure 4A). The root tip number (Figure 4C) and the root volume (Figure 4D) showed the same behavior, with values significantly increasing in wild-type plants under no P, but not in strigolactone-depleted plants. This trend was similar for root surface area, although with no significant differences in this case (Figure 4B). Differences were not significant for root diameter (Figure 4E), while lateral root number decreased significantly in the strigolactone-depleted plants at zero P reinforcing a non-significant trend observed also in wild-type plants (Figure 4F). This suggests that acclimation to zero P (in terms of greater root volume and higher lateral root number) is promoted by a period of growth on low P, and that such acclimation process is favored by strigolactones.

### 2.4. Strigolactone Depletion Alters Root Tip Morphology and Anatomy under Different P Availability

**Figure 5:**
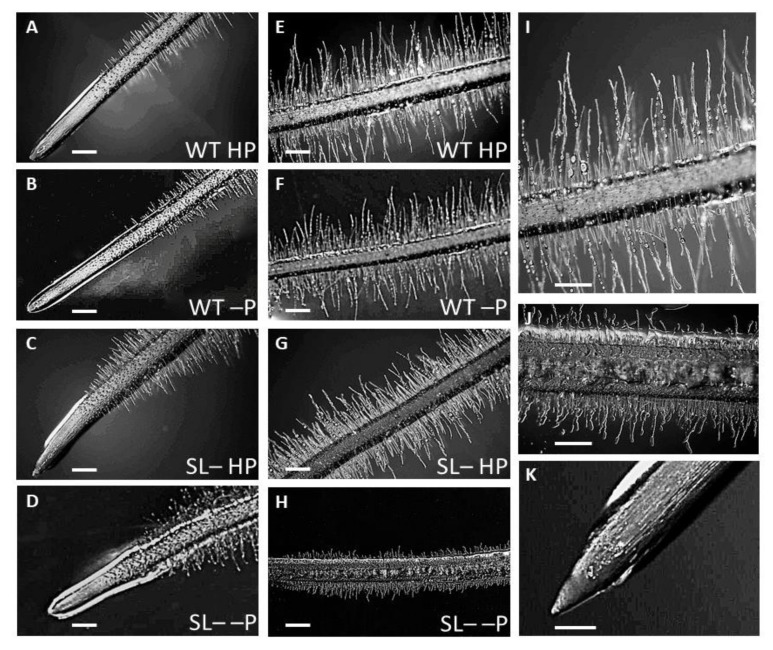
Root tip morphology of wild-type (WT) and strigolactone-depleted plants (SL–) grown for 10 days in MS medium with either high P or low P and further transferred for one week to MS medium containing either high P (HP) or no P (–P), respectively. (**A–D**) Root primary morphology, differentiation zone and tip of plants: (**A**) WT under HP (**B**) WT under –P (**C**) SL– under HP (**D**) SL– under –P. (**E–H**) zoom-in on the root differentiation zone of plants: (**E**) WT under HP (**F**) WT under –P (**G**) SL– under HP (**H**) SL– under –P. (**I**) Higher magnification of WT HP differentiated root primary structure; (**K**) Higher magnification of SL– –P differentiated root primary structure; (**K**) Higher magnification of SL–HP root tip. Scale bars: 200 µm (**A–H**); 1 mm (**I–K**).

As strigolactone depletion was shown to reduce root growth under low P availability, we hypothesized that this may be linked to anatomical modifications. We thus analyzed root tip morphology and anatomy in wild-type and strigolactone-depleted plants grown under the same experimental set-up as in the previous experiment, i.e., either with high P or no P supply, in the latter case after transfer from low P (acclimation period). The root tips of the wild-type plants were similar in shape, irrespective of P supply (Figure 5A,B), with abundant root hairs along the differentiation zone without visible differences in length (Figure 5E,F). However, wild-type plants seemingly produced more root rhizodeposition at the area of cell division and along the elongation zone when grown at zero P after an acclimation period on low P than when kept under high P conditions. This is suggested by the more abundant rhizodeposition on the fine hairs of wild-type plants under zero vs. high P (Figure 5A,B and Figure S1).

In strigolactone-depleted plants, the undifferentiated zone of the root tip was shorter compared to wild-type plants, with a different shape compared to wild-type plants and depending on P supply (Figure 5A–D,K). Specifically, the root tip was clearly cone-shaped in strigolactone-depleted plants under high P (Figure 5C,K), but club-shaped and rounder under zero P (Figure 5D). Under zero P, the root tip in strigolactone-depleted plants was abundantly coated by root exudates and displayed shorter root hairs (Figure 5H,J) compared with plants gown at high P (Figure 5G) and with wild-type plants under either P condition (Figure 5E,F,I). Conversely, root hairs in strigolactone-depleted plants were only slightly shorter compared to wild-type plants under high P conditions, but their density seemed to be higher than in the wild-type under any condition and especially under zero P.

**Figure 6:**
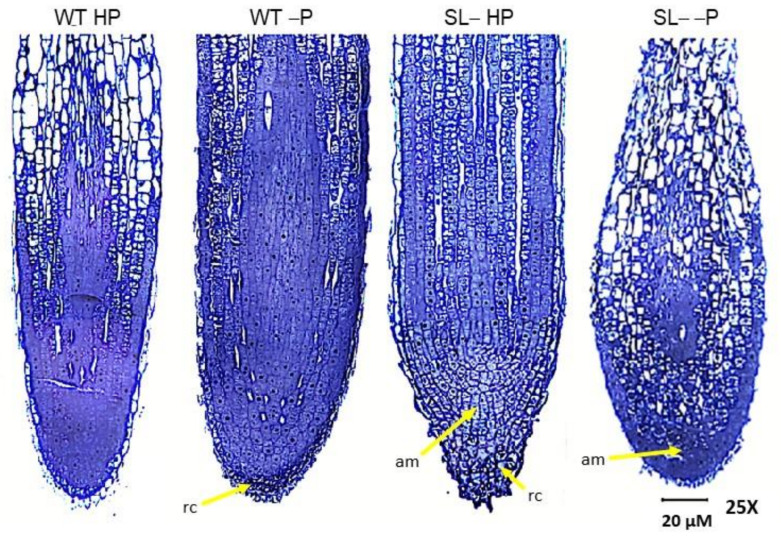
Light micrographs of longitudinally sectioned root tips of wild-type (WT) and strigolactone-depleted plants (SL–) grown for 10 days in MS medium with either high P or low P and further transferred for one week to MS medium containing either high P (HP) or no P (–P), respectively. In WT HP plants, the postmitotic isodiametric growth zone is reduced in length compared to WT –P plants, and the cells acquire an elongated shape closer to the apical meristem (am). In WT –P plants, an extended postmitotic isodiametric growth, with cells maintaining their isodiametric shape, is visible throughout the root tip. Note the wider root tip diameter compared to WT HP plants. In SL– HP plants, note the root cap (rc) with cells enclosing several small vacuoles, the cone shape of the root apex, and the maintenance of cells with meristematic features along the root longitudinal axis. Above the apical meristem are many cells with isodiametric shape and several vacuoles scattered in the cytoplasm. The root tip of SL– –P plants is swollen and displays high levels of disorganization. A small apical meristem is visible in the center. Above it, most cells are elongated, with a large central vacuole. Below it, cells are isodiametric with several small vacuoles.

**Figure 7:**
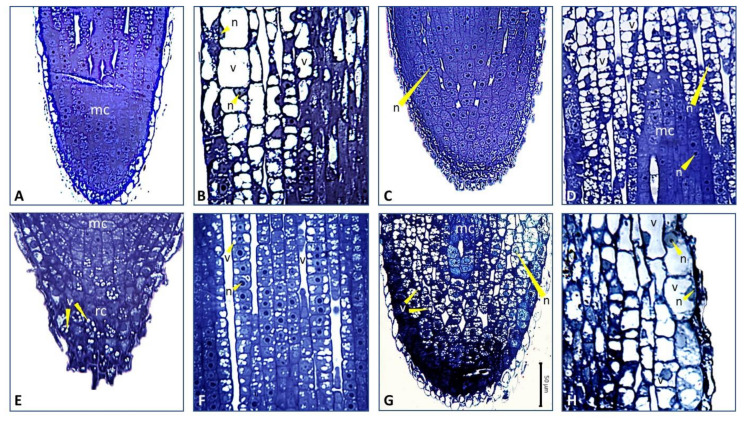
Light micrographs of selected areas of longitudinally sectioned root tips of wild-type (WT) and strigolactone-depleted (SL–) plants grown as in Figure 5 and Figure 6. High magnification images of (**A**) the root apex (mc: meristematic cells) and (**B**) elongation/differentiation zones of WT HP plants (about 100 µm above the apical meristem); (**C**) the root apex and (**D**) elongation zone (about 200 µm above the apical meristem) of WT –P plants. Cells have a big central nucleus (n), isodiametric shape, and some are still dividing; (**E**) the root cap (rc) and (**F**) elongation zone (about 200 µm above the apical meristem) of SL– HP plants. Note the presence of numerous vacuoles (v). The cells show very juvenile characteristics, with large nuclei and numerous small vacuoles scattered in the cytoplasm; (**G**) the root apex of SL– –P plants. Note the small cells enclosing numerous vacuoles in the root cap and below the root apex; (**H**) the area about 100 µm above the apical meristem in SL– –P plants. Note the transition from cells with several vacuoles to cells bigger in size with one central vacuole.

These observations were further confirmed by transmission light microscopy analyses (Figure 6, Figure 7). The root tips of wild-type plants grown under high P conditions showed the expected anatomical organization (Figure 6). The meristematic apex at the root tip consisted of small cells with isodiametric shape and a large nucleus in the center. The meristematic zone was longitudinally shorter compared to that observed at the root tip of P-starved wild-type plants. In the elongation zone, cells showed juvenile characteristics with diffuse vacuolization suggesting ongoing differentiation processes. In the differentiation zone, cells larger in size were evident, each one enclosing a prominent central vacuole, which is a typical feature of adult cells (Figure 6, Figure 7B). Phosphorus starvation caused anatomical changes in the root tips of wild-type plants, such as the extension of the meristematic zone along the longitudinal axis. This zone was, however, well organized, characterized by typical small cells, each one with a large nucleus in the centre (Figure 6, Figure 7C,D). In the elongation zone, cells maintained the nucleus in central position with several vacuoles scattered in the cytoplasm, which is typical of cells that are differentiating, but are not yet adult. The formation of large adult cells containing one or few vacuoles was clearly delayed compared to wild-type plants under high P conditions.

In roots of strigolactone-depleted plants supplied with high P, the tip and meristematic apex were cone-shaped, consistent with stereomicroscopy evidence (Figure 5K, Figure 6), and a number of cells in the cap contained several small vacuoles (Figure 6, Figure 7E). Also, numerous cells displayed isodiametric shape, large central nucleus and intense vacuolization above the meristematic apex, along the root longitudinal axis (Figure 6, Figure 7F). As observed for wild-type plants under no P, the acquisition of adult cell features was delayed compared to wild-type under full P supply. Finally, the root tips of strigolactone-depleted plants swell under no P, consistently with the club-shaped morphology observed under stereomicroscopy, and showed significant signs of tissue disorganization (Figure 6, Figure 7G). At the level of the meristematic apex and cap, cells contained a central nucleus and several small vacuoles, showing signs of differentiation. Above them, several more cells showed diffused vacuolization. Also, a restricted group of cells with meristematic traits was visible, while the root cap was inconspicuous. The differentiation zone, which is formed by typical adult cells, was maintained (Figure 6, Figure 7H).

## 3. Discussion

In this study, we investigated the effects of strigolactones on root growth and architecture, morphology and anatomy by comparing wild-type and strigolactone-treated or strigolactone-depleted tomato plants under different P supplies. We first examined the effects of the exogenous application of *rac*-GR24 on root development of wild-type plants grown under either P-sufficiency or low P. Then, we assayed the effects of impaired strigolactone synthesis on architecture and anatomy of roots adjusting to different P nutrition supply during growth.

*rac*-GR24 is the most widely used synthetic strigolactone analogue, with similar biological activity to that of endogenous strigolactones [8,37]. In this study, *rac*-GR24 application to wild-type tomato plants grown under P-limiting conditions increased the primary root length and number of lateral roots, and favored biomass allocation to the roots (Figure 1). Conversely, wild-type plants grown at high P were apparently not sensitive to exogenous *rac*-GR24 with respect to such traits. *rac*-GR24-induced root elongation was previously observed in tomato [35] and *A. thaliana* [38] plants, and was ascribed to the increase in number and length of cells located in the root meristem and transition zone, according to the auxin status of the plants [38,39]. In *A. thaliana*, primary root elongation was accompanied with a decrease of lateral root density and delayed development [14,30,38]. In these studies, strigolactones were proposed to act as modulators of the auxin flux, thus altering the auxin optima for lateral root formation. Therefore, under low P conditions, changes in root system architecture were attributed to increased sensitivity to auxin [38,39]. Interestingly, *rac*-GR24 application was reported to determine not completely overlapping effects in P-deprived rice plants, where it decreases primary root length and increases lateral root density [40]. However, it is noteworthy that P shortage inversely modulates these parameters in rice compared to *A. thaliana*, by increasing primary root length and reducing lateral root density [40,41,42]. In both species, variation in primary root length and lateral root density were ascribed to the (either promoting or inhibiting) effects of strigolactones on auxin transport within the root [39,40,43]. In our study, we suppose that exogenous *rac*-GR24 may have acted in roots of tomato plants via a crosstalk with auxin similarly to other species, and we suggest that, in tomato, strigolactones contribute to adjust those root traits that may favor soil exploration under low P availability. Additional mechanisms governing strigolactones effects on root development of tomato plants growing under P shortage, including the interaction of strigolactones with key molecular players in the phosphate starvation response and/or other phytohormones than auxins, cannot be excluded [1,11,44]. As a final note on this subset of data, it is worth noting that the racemic mixture we employed contains two stereoisomers, one of which can stimulate the KAI2-dependent pathway in *Arabidopsis* [45]. This same pathway was recently proven to influence specific root traits in this plant [11], with a possible confounding effect that is, however, still unproven in tomato.

The important role of strigolactones in modifying root traits of tomato plants during acclimation to P limiting conditions was also confirmed by the comparison between wild-type and strigolactone-depleted plants grown with high P, no P, or low P levels. Plants continuously grown under no P since soon after germination, as in the X-ray CT experiment (Figure 2), had an overall less developed root system; the typical responses to sudden or gradual P stress, such as decreased length of primary root, increased lateral root length and topsoil foraging [21,46,47] were not visible in either genotype. This apparent lack of response to nutritional stress both in the wild-type and the strigolactone-depleted plants may be due to the fact that full root growth could not be supported in the complete absence of P, hence preventing also meaningful morphological adjustments to stress in the wild-type [25]. When wild-type plants were grown under low P conditions, instead, their total root length and tip number were increased compared to wild-type plants supplied with high P, as expected; such response was not displayed by strigolactone-depleted plants. Our experiments also indicate that, in tomato, low strigolactone synthesis affects the ability of roots to respond to more gradual P decrease when plants are transferred from low P to no P, i.e., when they are allowed to acclimate before being exposed to complete P deprivation (Figure 4). Under these settings, a significant decrease in total root length was observed in P-starved strigolactone-depleted plants compared to both strigolactone-depleted plants and wild-type plants receiving high P. This decline was not due to less primary root elongation, but rather to a decline in lateral root number and length. These results provide further evidence of the role of strigolactones as regulators of lateral root formation and development, consistent with observations reported in the *A. thaliana* strigolactone-biosynthesis mutant *max4-1* [14]. As for strigolactone-dependent morphological responses at the root level reported in other plant species [38,43], these may happen via the modulation of auxin fluxes and localized auxin levels along the root axis of tomato as well.

Root hair development is under hormonal control [48] and increasing the amount of root hairs is a common strategy adopted by P-deprived plants to enhance the capacity of their roots to explore the rhizosphere for P scavenging [16,49]. A strigolactone-auxin crosstalk has been proposed to regulate root hair formation and elongation, with strigolactones triggering the increase in auxin accumulation in root epidermal cells through modulation of auxin flux from the root [9,26,39,50]. However, these reports were reassessed recently, leading to the conclusion that the sibling pathway initiated by KAI2 is instead responsible for root hair elongation in *Arabidopsis* [11]. Whether this holds true in tomato as well has not been addressed. In this work, we observed that, in addition to the reduction of lateral root growth (a trait that was confirmed to depend on strigolactones in *Arabidopsis*), strigolactone-depleted plants grown under P starvation exhibit a dramatic decrease in root hair elongation compared to the wild-type (Figure 5). Thus, we propose that lower strigolactone levels in tomato roots prevent root hair elongation under P deficiency, possibly by altering auxin levels in epidermal cells. It is noteworthy that, although root tips of strigolactone-depleted, P-sufficient plants were characterized by only slightly shorter root hairs compared to wild-type plants, their density was apparently higher.

If, on the one hand, root hairs are important to increase the root surface area and the portion of soil explored by roots, on the other hand root tips are of primary importance in nutrient sensing. The physical contact with low-P media is necessary to reprogram the whole root architecture [51,52], with inorganic P itself acting as a signaling molecule [53]. Therefore, analyzing changes in root tip morphology and anatomy could help in elucidating the overall plant response to nutrient stress. Clear alterations of root tip anatomy in strigolactone-depleted, P-starved plants were visible (Figure 6 and Figure 7), which may explain why these plants were less efficient in developing their roots under P starvation when compared to the wild-type. The root tip was indeed characterized by extensive cell and tissue disorganization, possibly due to unbalanced levels not only of strigolactones, but also other hormones known to control cell division and differentiation processes at the root apex [17]. Tomato seems hypersensitive to P-limitation stress when strigolactone biosynthesis is reduced. This hypothesis is supported by the observation that, in P-sufficient plants, low strigolactones caused moderate anatomical changes in the root tip, which were similar to those observed in wild-type plants shifted to no P supply after acclimation at low P. In the latter group, the root meristem was more developed than in wild-type controls under adequate P, thus indicating that division processes of wild-type plants were not affected by P deficiency under our experimental conditions, at least at the root apex. Additionally, the processes of cell differentiation and maturation were clearly delayed compared to wild-type plants kept under high P. Instead, the root apex of strigolactone-depleted plants was markedly altered under P stress. Low strigolactone biosynthesis along with P starvation, and complex cross-talks of strigolactones with other hormone pathways could be responsible for such observed alterations. Strigolactones indeed proved to promote crown root elongation by stimulating meristematic cell division, via modulation of the local auxin concentrations controlling meristem cell number [42]. Auxins could further interact with the cytokinin signaling pathway that impacts stem cells patterning. The overall shape was different and peculiarly club-shaped; the internal anatomy showed some hallmarks of the determinate developmental reprogramme that is induced by P starvation in *Arabidopsis* [54]. Previous studies have shown indeed that the rates of cell division at the root meristem and of root cell elongation decrease in *A. thaliana* with decreasing P availability, and concomitantly the number of cells within the elongation zone is reduced while precocious differentiation and meristem reduction is observed [55].

The activity of meristems within a plant is tightly coordinated to optimize root growth in response to environmental conditions and many mobile signals, including auxin, cytokinins, and possibly strigolactones can modulate cell growth and differentiation, as well as meristem shape [17,56]. Interestingly, strigolactone-depleted plants under P-replete conditions exhibited a cone-shaped root tip, coated by a prominent root cap formed by cells with diffuse vacuolization, which could contribute to rhizodeposition. Intense rhizodeposition in strigolactone-depleted plants was most pronounced under P starvation, where root tips presented not only vacuole-rich cells in the root cap but also below/around the apical meristem. Rhizodeposition seemingly was also greater in wild-type plants in P deprivation compared to non-stressed wild-type, as revealed by stereomicroscopy.

Despite the effects of defective strigolactone production combined with P starvation being clear and consistent at the root level, differences between genotypes were in general not significant under adequate P conditions. These results suggest that, at least when abundant P is available, strigolactone-depleted plants maintain their capacity to acquire P from the external medium to sustain their growth.

## 4. Materials and Methods

### 4.1. Plant Material

In this study, tomato (*Solanum lycopersicum* L.) M82 was used (wild-type) and contrasted with line 6936, hereafter called strigolactone-depleted [34]. In this genotype, the key strigolactone-biosynthetic gene *SlCCD7* is knocked down by RNAi; production of the major strigolactones is thus reduced by about 80%–90% with respect to its wild-type M82. Both strigolactone-depleted and M82 plants have been previously characterized in terms of strigolactone biosynthesis, shoot branching and mycorrhiza-induced apocarotenoid formation [34]. Plants were grown under different P conditions and using different substrates, depending on the type of analysis performed, as described below. In all experiments, seeds were surface sterilized in 70% (v/v) ethanol for 2 min, then in 3% sodium hypochlorite for 20 min and washed five times for 10 min with deionized water. Unless otherwise stated, seeds were pre-germinated on wet Whatman filter paper in Petri dishes (10 cm diameter) inside a growth chamber at 22 °C and in the dark for 4 days and then grown in growth chambers with a 16/8 h light/dark cycle, air temperature of 22 °C and 50%–75% relative humidity with a light intensity of 100 µmol m^−2^ s^−1^. P concentrations were chosen based on preliminary results showing them to cause the most pronounced differences under each experimental set-up.

### 4.2. Root System Architecture Changes in Response to Exogenous Application of the Synthetic Strigolactone Analogue rac-GR24

The dependence of root architecture features on strigolactone availability was studied in tomato seedlings grown in vitro. Wild-type seedlings were grown in square Petri dishes (12 × 12 cm) containing either a full Murashige and Skoog (MS) medium [57] as a positive control (high P, 625 µM) or a modified MS medium with low levels of KH_2_PO_4_ (low P, 6.25 µM). For each P condition, the synthetic strigolactone analogue *rac*-GR24 was dissolved in 0.1% acetone at 5 µM final concentration (strigolactone-treated groups) while comparable amounts of acetone solution were added to the control groups for mock treatment. Pre-germinated seeds of wild-type plants were sown (6 seeds per plate, 1 plate per treatment; each seedling a replicate) and the Petri dishes were placed vertically in a walk-in growth chamber. Two weeks after sowing, the length of the primary root and the number of lateral roots were evaluated by scanning the plates and analyzing the images using the software ImageJ. Fresh shoot and root biomass were also quantified at the end of the trials. To confirm results, this experiment was repeated twice.

### 4.3. Root System Architecture Phenotyping of Wild-Type and Strigolactone-depleted Plants Grown under Continuously High or No P Conditions

After germination, wild-type and strigolactone-depleted seeds were transferred to columns (5 cm diameter × 12 cm height) containing quartz sand (<1 mm) and placed inside a growth chamber (Conviron A1000, Canada). Plants were watered daily with a modified Hoagland nutrient solution containing either 80 µM KH_2_PO_4_ (high P) or no KH_2_PO_4_ (no P). After 10 days of growth, each column was placed into the scanner (GE v|tome|x M 240 kV) and scanned using X-ray energy settings of 140 kV and 160 µA, in “FAST” mode. Three individual scans were required to image the entire column depth at a resolution of 35 µm. Scanned radiograph images were then reconstructed and combined in DatosX REC software (GE Measurement & Control, Germany) and 3D images were visualized with VGStudioMax v2.0 (Volume Graphics GmbH, Germany). The following root traits were recorded: total root length, primary root length, root surface area, root volume, average lateral root length, root tip number (indicative of total root number). Root trait data were obtained with Rooth software [58].

### 4.4. Root System Architecture Phenotyping of Wild-Type and Strigolactone-Depleted Plants Grown under Increasing Levels of P Starvation

To evaluate the effect of increasing P nutritional stress on both root system architecture and anatomy of wild-type and strigolactone-depleted tomato plants, pre-germinated seeds of the two lines were transferred to square Petri dishes (10 × 10 cm) filled with a modified half-strength MS medium [57], containing either high (80 µM) or low (10 µM) KH_2_PO_4_, at a density of 5 seeds per plate, and allowed to grow for 10 days inside a growth chamber. Representative wild-type and strigolactone-depleted plants grown under high or low P were then transferred for one week to sterile boxes (13 cm length × 20 cm height × 2 cm depth), at a density of 3 plants per box, containing the same MS medium as described previously, with either 80 µM KH_2_PO_4_ (high P) or no KH_2_PO_4_ (no P), respectively. Specifically, wild-type and strigolactone-depleted plants previously given with 80 µM of KH_2_PO_4_ were grown under the same P concentration, while wild-type and strigolactone-depleted plants initially treated with low P were transferred to MS medium without P. To obtain a full picture of the root system, root scanning was performed using an Epson Expression 10000XL 1.0 system (Regent Instruments Company, Canada) [59]. The following parameters were recorded with a root image analysis system using the software WinRHIZO: Root length (cm), surface area (cm^2^), volume (cm^3^), average diameter (mm), number of tips (referred to roots with a diameter < 2mm) and lateral roots (referred to roots with a length varying from 0 to 4.5 cm).

### 4.5. Stereo and Light Microscopy

Roots were further analyzed via microscopy. The root tip of wild-type and strigolactone-depleted plants initially grown with either high or low P and further transferred to either high P (80 µM KH_2_PO_4_) or no P (0 µM KH_2_PO_4_) were first subjected to observation under a stereo microscope (Leica Microsystems). Root tip segments were collected for additional analyses of root anatomy, fixed in 6% glutaraldehyde and processed for light microscopy as previously described [60]. Thin sections (1 µm thick) were cut with an Ultracut Reichert-Jung ultramicrotome, stained with 1% toluidine blue and 1% tetraborate (1:1, v/v), and observed and photographed under a Leitz Ortholux microscope.

### 4.6. Statistics

For all datasets, the analysis of variance (one-way ANOVA) was performed using the SPSS software version 18.0 (SPSS, Chicago, IL, USA), and was followed by pair-wise post-hoc analyses (Student–Newman–Keuls test) to determine which means differed significantly at *p <* 0.05 (± SD).

## 5. Conclusions

**Figure 8:**
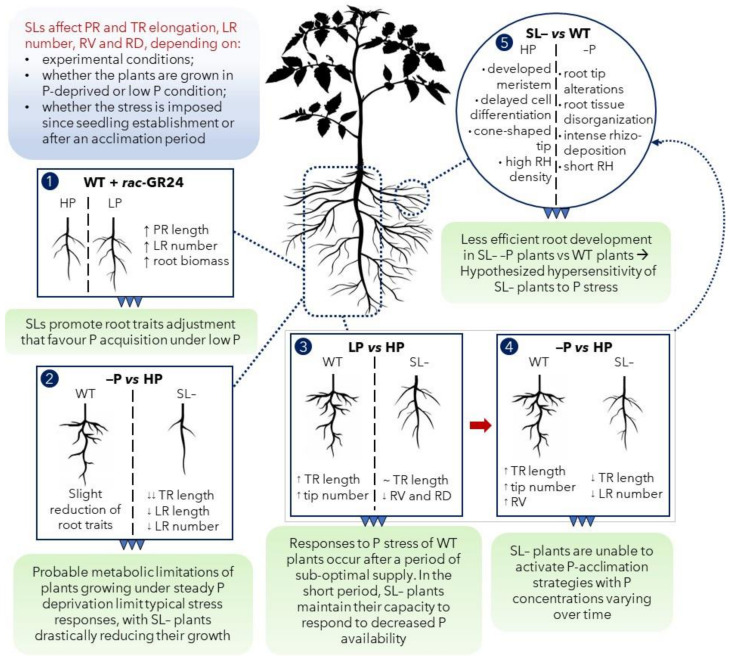
Conclusive conceptual summary. (**1**) Strigolactone (SL) analogue (*rac*-GR24) application to wild-type (WT) tomato plants under low P promoted root morphological modification to favor P acquisition; (**2**) the continuous growth under P stress (–P) limited root capacity to respond to P stress, especially in the strigolactone-depleted (SL–) plants; (**3**) under suboptimal P supply (LP), a detectable role of SL in improving plants responses to P stress was highlighted: SL– plants showed an attenuated capacity to respond to LP, for some specific features; (**4**) imposition of severe stress (no P, –P in the scheme) after acclimation on LP revealed the inability of SL– plants to respond to P stress in the long term, possibly because of root development impairment caused by the anatomical modifications observed at the microscopic scale (**5**). Abbreviations: PR: primary root; TR: total root; LR: lateral root; RV: root volume; RD: root diameter; RH: root hair.

In conclusion, this work provides further evidence in support of the biological role of strigolactones in mediating plant acclimation responses to P nutritional levels, and presents new insights on their effects on the root system of tomato plants. Specifically, we show by pharmacological and genetic means that, depending on whether plants are grown under totally P-deprived conditions or in suboptimal P levels, strigolactones significantly affect certain root traits, such as primary and total root elongation, lateral root number, root volume and diameter, which all allow for enhanced soil exploration by the roots (Figure 8). In particular, possibly due to unsustainable metabolic limitations, complete P deprivation since germination impairs typical stress responses, with strigolactone-depleted plants drastically reducing their growth with respect to wild-type. A period of sub-optimal P supply instead induces responses to P stress in wild-type plants, while some features of these responses are attenuated in strigolactone-depleted plants. If P stress is imposed after an initial acclimation period, these trait modifications are emphasized in wild-type plants, while significantly repressed in strigolactone-deficient plants, similar to plants held under continuous P shortage. Phenotypic differences between the two genotypes were obvious both at the root morphology and especially at the tip anatomy levels: hypersensitivity to P deprivation stress was clearly observed in tips of strigolactone-depleted roots, in terms of cell differentiation and tissue specification.

## Supplementary Materials

The following are available online at https://www.mdpi.com/2223-7747/9/5/612/s1, Figure S1: Root rhizodeposition (r) at the root tip (**A**) and at the differentiation zone (**B**) of P starved strigolactone-depleted plants. Note the short root hairs.

## References

[1] Koltai H. (2013). Strigolactones activate different hormonal pathways for regulation of root development in response to phosphate growth conditions. Ann. Bot..

[2] Umehara M. (2011). Strigolactone, a key regulator of nutrient allocation in plants. Plant Biotechnol..

[3] Xie X., Yoneyama K., Yoneyama K. (2010). The Strigolactone Story. Annu. Rev. Phytopathol..

[4] Liu W., Kohlen W., Lillo A., den Camp R.O., Ivanov S., Hartog M., Limpens E., Jamil M., Smaczniak C., Kaufmann K. (2011). Strigolactone biosynthesis in *Medicago truncatula* and rice requires the symbiotic GRAS-type transcription factors NSP1 and NSP2. Plant Cell.

[5] Alder A., Jamil M., Marzorati M., Bruno M., Vermathen M., Bigler P., Ghisla S., Bouwmeester H., Beyer P., Al-Babili S. (2012). The path from β-carotene to carlactone, a strigolactone-like plant hormone. Science.

[6] Cook C., Whichard L.P., Turner B., Wall M.E., Egley G.H. (1966). Germination of witchweed (*Striga lutea* Lour.): Isolation and properties of a potent stimulant. Science.

[7] Akiyama K., Matsuzaki K., Hayashi H. (2005). Plant sesquiterpenes induce hyphal branching in arbuscular mycorrhizal fungi. Nature.

[8] Umehara M., Hanada A., Yoshida S., Akiyama K., Arite T., Takeda-Kamiya N., Magome H., Kamiya Y., Shirasu K., Yoneyama K. (2008). Inhibition of shoot branching by new terpenoid plant hormones. Nature.

[9] Czarnecki O., Yang J., Weston D.J., Tuskan G.A., Chen J.-G. (2013). A dual role of strigolactones in phosphate acquisition and utilization in plants. Int. J. Mol. Sci..

[10] Cardinale F., Korwin Krukowski P., Schubert A., Visentin I. (2018). Strigolactones: Mediators of osmotic stress responses with a potential for agrochemical manipulation of crop resilience. J. Exp. Bot..

[11] Villaécija-Aguilar J.A., Hamon-Josse M., Carbonnel S., Kretschmar A., Schmid C., Dawid C., Bennett T., Gutjahr C. (2019). SMAX1/SMXL2 regulate root and root hair development downstream of KAI2-mediated signaling in Arabidopsis. PLoS Genet..

[12] Koltai H. (2011). Strigolactones are regulators of root development. New Phytol..

[13] López-Ráez J.A., Charnikhova T., Fernández I., Bouwmeester H., Pozo M.J. (2011). Arbuscular mycorrhizal symbiosis decreases strigolactone production in tomato. J. Plant Physiol..

[14] Mayzlish-Gati E., De-Cuyper C., Goormachtig S., Beeckman T., Vuylsteke M., Brewer P.B., Beveridge C.A., Yermiyahu U., Kaplan Y., Enzer Y. (2012). Strigolactones are involved in root response to low phosphate conditions in Arabidopsis. Plant Physiol..

[15] Vance C.P., Uhde-Stone C., Allan D.L. (2003). Phosphorus acquisition and use: Critical adaptations by plants for securing a nonrenewable resource. New Phytol..

[16] Aziz T., Sabir M., Farooq M., Maqsood M.A., Ahmad H.R., Warraich E.A., Hakeem K.R., Rehman R.U.I., Tahir I. (2014). Phosphorus deficiency in plants: Responses, adaptive mechanisms, and signaling. Plant Signaling: Understanding the Molecular Crosstalk.

[17] Niu Y.F., Chai R.S., Jin G.L., Wang H., Tang C.X., Zhang Y.S. (2013). Responses of root architecture development to low phosphorus availability: A review. Ann. Bot..

[18] Abel S., Ticconi C.A., Delatorre C.A. (2002). Phosphate sensing in higher plants. Physiol. Plant..

[19] Schachtman D.P., Reid R.J., Ayling S.M. (1998). Phosphorus uptake by plants: From soil to cell. Plant Physiol..

[20] Holford I.C.R. (1997). Soil phosphorus: Its measurement, and its uptake by plants. Soil Res..

[21] Péret B., Clément M., Nussaume L., Desnos T. (2011). Root developmental adaptation to phosphate starvation: Better safe than sorry. Trends Plant Sci..

[22] Shen J., Yuan L., Zhang J., Li H., Bai Z., Chen X., Zhang W., Zhang F. (2011). Phosphorus dynamics: From soil to plant. Plant Physiol..

[23] Hinsinger P. (2001). Bioavailability of soil inorganic P in the rhizosphere as affected by root-induced chemical changes: A review. Plant Soil.

[24] Santoro V., Martin M., Persson P., Lerda C., Said-Pullicino D., Magnacca G., Celi L. (2019). Inorganic and organic P retention by coprecipitation during ferrous iron oxidation. Geoderma.

[25] Wissuwa M., Gamat G., Ismail A.M. (2005). Is root growth under phosphorus deficiency affected by source or sink limitations?. J. Exp. Bot..

[26] López-Bucio J., Cruz-Ramırez A., Herrera-Estrella L. (2003). The role of nutrient availability in regulating root architecture. Curr. Opin. Plant Biol..

[27] Schroeder M.S., Janos D.P. (2005). Plant growth, phosphorus nutrition, and root morphological responses to arbuscular mycorrhizas, phosphorus fertilization, and intraspecific density. Mycorrhiza.

[28] Shen Q., Wen Z., Dong Y., Li H., Miao Y., Shen J. (2018). The responses of root morphology and phosphorus-mobilizing exudations in wheat to increasing shoot phosphorus concentration. AoB Plants.

[29] Umehara M., Hanada A., Magome H., Takeda-Kamiya N., Yamaguchi S. (2010). Contribution of strigolactones to the inhibition of tiller bud outgrowth under phosphate deficiency in rice. Plant Cell Physiol..

[30] Kapulnik Y., Delaux P.M., Resnick N., Mayzlish-Gati E., Wininger S., Bhattacharya C., Séjalon-Delmas N., Combier J.P., Bécard G., Belausov E. (2011). Strigolactones affect lateral root formation and root-hair elongation in *Arabidopsis*. Planta.

[31] López-Ráez J.A., Charnikhova T., Gómez-Roldán V., Matusova R., Kohlen W., De Vos R., Verstappen F., Puech-Pages V., Bécard G., Mulder P. (2008). Tomato strigolactones are derived from carotenoids and their biosynthesis is promoted by phosphate starvation. New Phytol..

[32] Kohlen W., Charnikhova T., Bours R., López-Ráez J.A., Bouwmeester H. (2013). Tomato strigolactones: A more detailed look. Plant Signal Behav..

[33] Rial C., Varela R.M., Molinillo J.M.G., López-Ráez J.A., Macías F.A. (2019). A new UHPLC-MS/MS method for the direct determination of strigolactones in root exudates and extracts. Phytochem. Anal..

[34] Vogel J.T., Walter M.H., Giavalisco P., Lytovchenko A., Kohlen W., Charnikhova T., Simkin A.J., Goulet C., Strack D., Bouwmeester H.J. (2010). SlCCD7 controls strigolactone biosynthesis, shoot branching and mycorrhiza-induced apocarotenoid formation in tomato. Plant J..

[35] Koltai H., Lekkala S.P., Bhattacharya C., Mayzlish-Gati E., Resnick N., Wininger S., Dor E., Yoneyama K., Hershenhorn J., Joel D.M. (2010). A tomato strigolactone-impaired mutant displays aberrant shoot morphology and plant interactions. J. Exp. Bot..

[36] Kohlen W., Charnikhova T., Lammers M., Pollina T., Tóth P., Haider I., Pozo M.J., de Maagd R.A., Ruyter-Spira C., Bouwmeester H.J. (2012). The tomato *CAROTENOID CLEAVAGE DIOXYGENASE 8* (*SlCCD8*) regulates rhizosphere signaling, plant architecture and affects reproductive development through strigolactone biosynthesis. New Phytol..

[37] Gomez-Roldan V., Fermas S., Brewer P.B., Puech-Pagès V., Dun E.A., Pillot J.P., Letisse F., Matusova R., Danoun S., Portais J.C. (2008). Strigolactone inhibition of shoot branching. Nature.

[38] Ruyter-Spira C., Kohlen W., Charnikhova T., van Zeijl A., van Bezouwen L., de Ruijter N., Cardoso C., Lopez-Raez J.A., Matusova R., Bours R. (2011). Physiological effects of the synthetic strigolactone analog GR24 on root system architecture in Arabidopsis: Another belowground role for strigolactones?. Plant Physiol..

[39] Koltai H., Dor E., Hershenhorn J., Joel D.M., Weininger S., Lekalla S., Shealtiel H., Bhattacharya C., Eliahu E., Resnick N. (2010). Strigolactones’ effect on root growth and root-hair elongation may be mediated by auxin-efflux carriers. J. Plant Growth Regul..

[40] Sun H., Tao J., Liu S., Huang S., Chen S., Xie X., Yoneyama K., Zhang Y., Xu G. (2014). Strigolactones are involved in phosphate- and nitrate-deficiency-induced root development and auxin transport in rice. J. Exp. Bot..

[41] Pérez-Torres C.-A., López-Bucio J., Cruz-Ramírez A., Ibarra-Laclette E., Dharmasiri S., Estelle M., Herrera-Estrella L. (2008). Phosphate availability alters lateral root development in *Arabidopsis* by modulating auxin sensitivity via a mechanism involving the TIR1 auxin receptor. Plant Cell.

[42] Arite T., Kameoka H., Kyozuka J. (2012). Strigolactone positively controls crown root elongation in rice. J. Plant Growth Regul..

[43] Sun H., Xu F., Guo X., Wu D., Zhang X., Lou M., Luo F., Zhao Q., Xu G., Zhang Y. (2019). A Strigolactone signal inhibits secondary lateral root development in rice. Front. Plant Sci..

[44] Ma Z., Baskin T.I., Brown K.M., Lynch J.P. (2003). Regulation of root elongation under phosphorus stress involves changes in ethylene responsiveness. Plant Physiol..

[45] Scaffidi A., Waters M.T., Sun Y.K., Skelton B.W., Dixon K.W., Ghisalberti E.L., Flematti G.R., Smith S.M. (2014). Strigolactone hormones and their stereoisomers signal through two related receptor proteins to induce different physiological responses in Arabidopsis. Plant Physiol..

[46] Péret B., Desnos T., Jost R., Kanno S., Berkowitz O., Nussaume L. (2014). Root architecture responses: In search of phosphate. Plant Physiol..

[47] Lynch J.P. (2011). Root phenes for enhanced soil exploration and phosphorus acquisition: Tools for future crops. Plant Physiol..

[48] Omoarelojie L.O., Kulkarni M.G., Finnie J.F., Van Staden J. (2019). Strigolactones and their crosstalk with other phytohormones. Ann. Bot..

[49] Lynch J.P. (2007). Roots of the second green revolution. Aust. J. Bot..

[50] Kapulnik Y., Resnick N., Mayzlish-Gati E., Kaplan Y., Wininger S., Hershenhorn J., Koltai H. (2011). Strigolactones interact with ethylene and auxin in regulating root-hair elongation in *Arabidopsis*. J. Exp. Bot..

[51] Svistoonoff S., Creff A., Reymond M., Sigoillot-Claude C., Ricaud L., Blanchet A., Nussaume L., Desnos T. (2007). Root tip contact with low-phosphate media reprograms plant root architecture. Nat. Gen..

[52] Abel S. (2011). Phosphate sensing in root development. Curr. Opin. Plant Biol..

[53] Ticconi C.A., Delatorre C.A., Abel S. (2001). Attenuation of phosphate starvation responses by phosphite in Arabidopsis. Plant Physiol..

[54] Sánchez-Calderón L., López-Bucio J., Chacón-López A., Cruz-Ramírez A., Nieto-Jacobo F., Dubrovsky J.G., Herrera-Estrella L. (2005). Phosphate Starvation Induces a Determinate Developmental Program in the Roots of *Arabidopsis thaliana*. Plant Cell Physiol..

[55] Rouached H., Arpat A.B., Poirier Y. (2010). Regulation of phosphate starvation responses in plants: Signaling players and cross-talks. Mol. Plant.

[56] Brewer P.B., Dun E.A., Gui R., Mason M.G., Beveridge C.A. (2015). Strigolactone inhibition of branching independent of polar auxin transport. Plant Physiol..

[57] Murashige T., Skoog F. (1962). A revised medium for rapid growth and bio assays with tobacco tissue cultures. Physiol. Plant..

[58] Mairhofer S., Pridmore T., Johnson J., Wells D.M., Bennett M.J., Mooney S.J., Sturrock C.J. (2017). X-ray computed tomography of crop plant Root systems grown in soil. Curr. Protoc. Plant Biol..

[59] Ding Y., Feng R., Wang R., Guo J., Zheng X. (2014). A dual effect of Se on Cd toxicity: Evidence from plant growth, root morphology and responses of the antioxidative systems of paddy rice. Plant Soil.

[60] Bonghi C., Casadoro G., Ramina A., Rascio N. (1993). Abscission in leaf and fruit explants of *Prunus persica* (L.) Batsch. New Phytol..

