# Supplementary material for: Strigolactones Control Root System Architecture and Tip Anatomy in Solanum lycopersicum L. Plants under P Starvation
Source: Plants (Basel). 2020 May 11;9(5):612. doi: 10.3390/plants9050612 (PMC7285494; doi:10.3390/plants9050612)
Supplement: Supplementary file 1 [file plants-09-00612-s001.pdf]

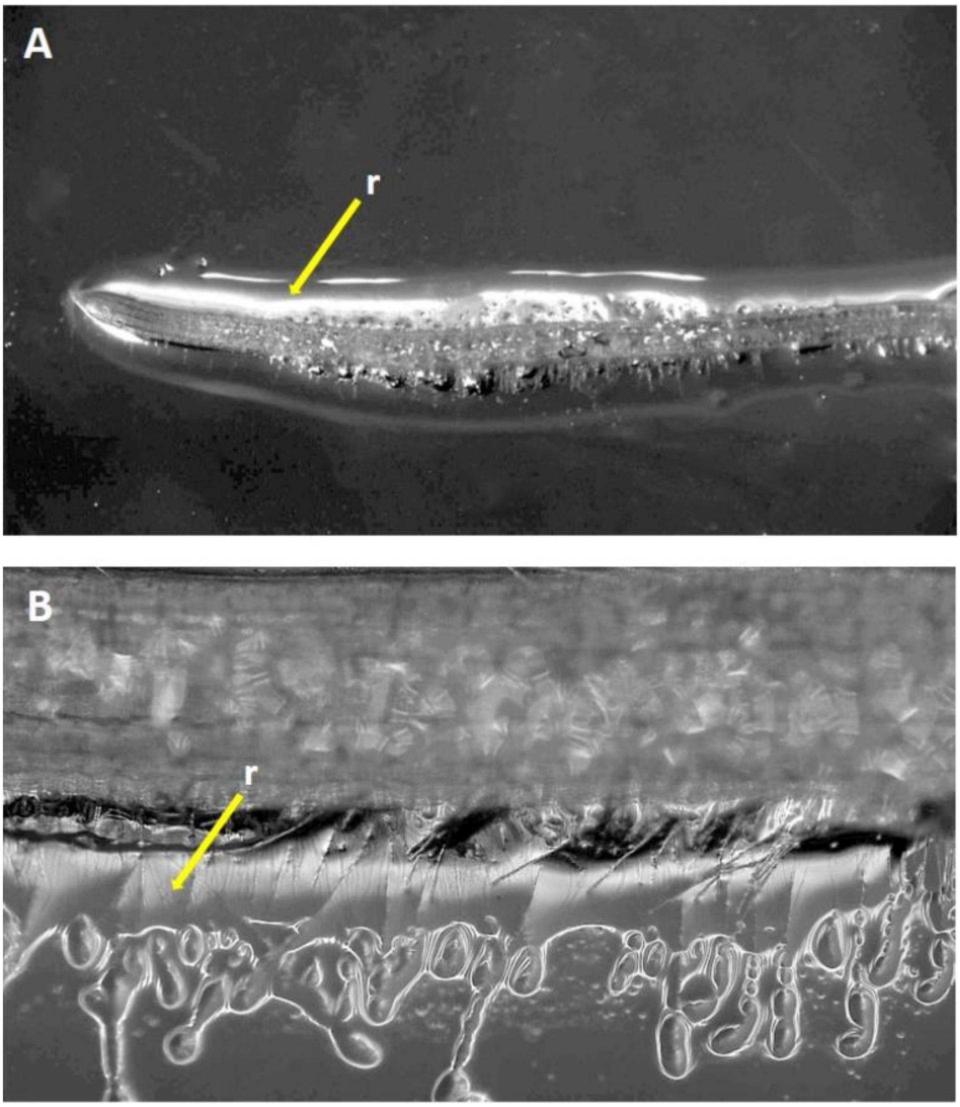


**Figure S1.** Root rhizodeposition (r) at the root tip (**A**) and at the differentiation zone (**B**) of P starved strigolactone-depleted plants. Note the short root hairs.
